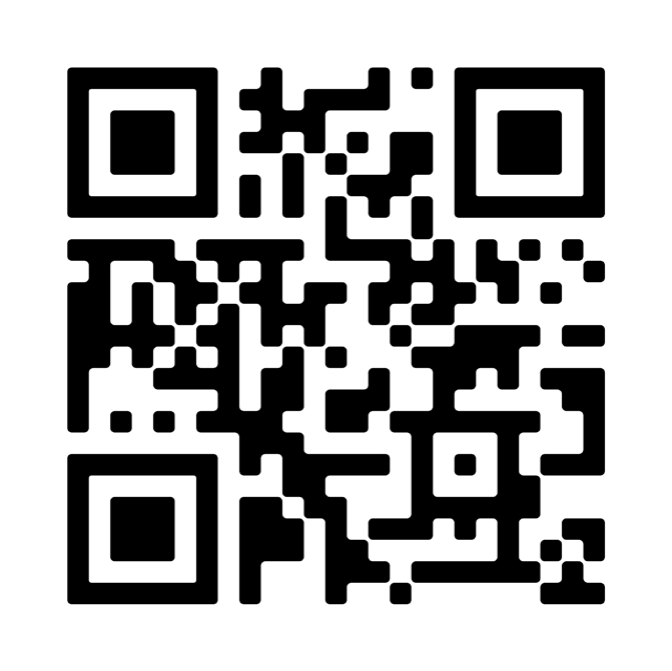# Caesarean scar defect and retained products of conception (RPOC): a step-by-step combined hysteroscopic and laparoscopic treatment

**DOI:** 10.52054/FVVO.16.3.031

**Published:** 2024-09-30

**Authors:** G Panico, S Mastrovito, E Bonetti, F Fanfani, G Scambia, U Catena

**Affiliations:** Fondazione Policlinico Universitario A. Gemelli IRCCS, UOC Ginecologia Oncologica, Dipartimento di Scienze della Salute della Donna e del Bambino e di Sanità Pubblica, Roma, Italia, 00168; Department of Clinical and Experimental Sciences, University of Brescia, 25136 Brescia, Italy, Università degli Studi di Brescia, Dipartimento di Scienze Cliniche e Sperimentali, ASST Spedali Civili di Brescia; Università Cattolica del Sacro Cuore, Fondazione Policlinico Universitario A. Gemelli IRCCS, UOC Ginecologia Oncologica, Dipartimento di Scienze della Salute della Donna e del Bambino e di Sanità Pubblica, Roma, Italia, 00168

**Keywords:** Laparoscopy, hysteroscopy, isthmocele, caesarean scar defect, isthmoplasty, Retained Products of Conception (RPOC)

## Abstract

**Background:**

Uterine scar defect (also called uterine niche or isthmocele) associated to retained products of conception (RPOC) is an uncommon occurrence following caesarean section. Typically, the primary indicator is abnormal vaginal bleeding, and an accurate diagnosis can be established through ultrasound evaluation. Several surgical and endoscopic treatments have been described.

**Objectives:**

To show a step-by-step video of combined hysteroscopic and laparoscopic approach to perform isthmocele repair in a patient with caesarean scar defect and RPOC.

**Materials and Methods:**

We report a case of a 34-year-old patient who was referred to our Digital Hysteroscopic Clinic (DHC) for abnormal vaginal bleeding and persistent pelvic pain, three months after a caesarean section. A single-step diagnostic approach through transvaginal ultrasound and diagnostic hysteroscopy revealed the presence of an isthmic uterine niche within the caesarean scar area, containing a poorly vascularised heterogeneous hyperechoic focal mass measuring 33x11x33 millimetres.

**Main outcome measures:**

Removal of RPOC and surgical complications.

**Results:**

All retained placental tissue was removed and the uterine wall defect was corrected. No complications occurred and the patient was discharged two days after the procedure. Patient was asymptomatic at 3 months follow up and ultrasound and hysteroscopy showed a reconstituted uterine wall.

**Conclusion:**

An integrated hysteroscopic and laparoscopic approach seems to be an effective conservative method to remove RPOC and perform isthmocele repair with optimal surgical results.

## Learning objective

To propose a conservative minimally invasive approach for isthmoplasty and removal of RPOC in a symptomatic patient affected by caesarean scar defect with placental remnants infiltrating the myometrium.

## Introduction

The caesarean scar defect is a rare occurrence following a caesarean section, often presenting with atypical vaginal bleeding as its primary symptom. The main factors contributing to uterine niche formation include inadequate wound healing, infection or trauma in the affected area (Mraihi et al., 2023). Another potential factor leading to post-partum haemorrhage is the presence of retained products of conception (RPOC), with a relatively low incidence, of around 1% (Capmas et al., 2019).

The diagnosis for both isthmocele and RPOC is usually based on ultrasound, which can identify the interrupted scar region and the presence of a heterogeneous hyperechoic and intracavitary focal mass. The use of colour Doppler examination can improve the accuracy of the diagnosis (Yagur et al. 2023). Possible approaches for the management of uterine defects include conservative as well as surgical options. The selection of the most appropriate strategy is determined by patient’s clinical status and the extent of the isthmocele. Several strategies have also been described for the management of RPOC, ranging from expectant or medical management to surgical treatment. Goldenberg et al. (1997) was the first to report the use of hysteroscopy for the removal of RPOC.

We present a case of major uterine scar defect with RPOC infiltrating the myometrium. The condition was diagnosed three months after caesarean delivery, after assessment of mild vaginal bleeding. To address both the isthmocele and RPOC in a single procedure, we introduce a “single-step” combined laparoscopic and hysteroscopic technique.

## Patients and methods 

A 34-year-old patient, G1P1, with no significant medical history, was referred to our outpatient clinic with a history of vaginal bleeding and pelvic pain. A caesarean section was performed three months prior in a different hospital, and there was no available information regarding occult placenta accreta. A transvaginal ultrasound suggested an isthmocele with an area of non-vascularised tissue measuring 33x11x33 millimetres protruding into the uterine niche, reaching the cervical canal. A diagnostic hysteroscopy confirmed the presence of retained placenta adherent to the anterior uterine wall, concealing the caesarean scar defect and occupying it almost completely.

We propose a step-by-step demonstration of a combined hysteroscopic and laparoscopic approach for the removal of the placental remnants and correction of the uterine scar defect. The procedure was performed in the division of Minimally Invasive Gynaecology, Fondazione Policlinico Gemelli IRCCS of Rome – Italy. The patient was given information on the different surgical approaches and was advised about the risks of the procedure. Signed informed consent, validated by our internal ethical committee, allowing the use of personal data was also given by the patient.

## Results

The procedure was carried out under general anaesthesia, as follows:

Using a 5-millimeters Bettocchi hysteroscope, access to the uterine cavity was obtained. The retained placental tissue was found occupying the anterior uterine cavity; the overall shape of the uterine cavity was regular, and the tubal ostia were visualised bilaterally.

Complete hysteroscopic removal of the intracavitary placental remnants was performed using Tissue Removal Device (TRD) (Truclear Elite Mini, Medtronic). After trans-umbilical laparoscopic access, dissection of the vesico-uterine space was performed to visualise the isthmus; under hysteroscopic transillumination, isthmic myometrium appeared interrupted, with only an extremely thin layer of placental remnants separating the uterine cavity from the abdominal cavity.

A hysterotomy was carried out, and the isthmic region was resected using a monopolar hook electrode, under precise hysteroscopic guidance with transillumination. The insertion of the hysteroscope into the cervical canal prevented canal occlusion during suturing of the defect, which was carried out through interrupted stitches with absorbable 1/0 thread (VICRYL™).

Final hysteroscopic assessment of the endometrial cavity confirmed a sufficiently thick and reinforced uterine wall, which was confirmed by a negative hydropneumatic test. All remaining placental tissue was successfully removed, and the myometrial defect was completely corrected. No intra and post-operative complications occurred, and blood loss was minimal. The patient was discharged on the second postoperative day. Histology results indicated inflammatory patterns. One month after surgery, the patient reported symptom resolution. Hysteroscopic and ultrasound follow-up at six months showed a normal uterine cavity with a regular anterior wall and no intrauterine adhesions or residues.

## Discussion

In this video article we propose a step-by-step description of a procedure for RPOC removal and correction of caesarean scar defect using a combined hysteroscopic and laparoscopic technique. This integrated treatment allowed for a single-step conservative approach in a young, fertile woman. Combined hysteroscopic morcellation with Tissue Removal Device (TRD) (Truclear Elite Mini, Medtronic) and laparoscopic resection of the infiltrated isthmic myometrium allowed for a complete removal of the placental remnants and a successful repair of the uterine defect.

The utilisation of tissue removal devices (TRD) has been extensively described for the treatment of intracavitary pathologies, including RPOC (Damiani et al., 2023). Compared to loop resection, this method seems to be associated with shorter operating time and lower risk of intrauterine adhesions (Hamerlynck et al., 2016).

During the isthmoplasty, hysteroscopic vision allowed the surgeon to identify the best site of uterine incision while preventing cervical canal occlusion, minimising risks of complications such as bladder injury which can be higher.

A combined hysteroscopic and laparoscopic approach for the treatment of symptomatic isthmocele has been already described using the “Rendez-Vous technique” (Nirgianakis et al., 2016). Recent literature suggests that hysteroscopic isthmoplasty is an effective and safe treatment option in cases where the isthmocele’s niche is deeper than 2 mm and there is a minimal residual myometrial thickness of 3 mm; if the residual myometrial thickness is less than 3 mm, the hysteroscopic approach is not recommended primarily due to the risk of bladder injury, and laparoscopic repair may a better option (Di Spiezio Sardo et al., 2018). In the wider literature, combined endoscopic approaches for the treatment of large symptomatic isthmoceles with minimal residual myometrium have been described, allowing for a more complete resection of scarred tissue; the light of the hysteroscope, through transillumination (“Halloween sign”), allows the laparoscopist to identify the edges of the scar (Vigueras Smith et al., 2020; Smet et al., 2023).

The strength of our technique lies in its ability to simultaneously address both uterine niche and RPOC in a single combined endoscopic approach, eliminating the need for multiple surgical interventions and achieving optimal surgical results. It is important to note that this approach demands a high level of surgical proficiency and should be conducted by skilled operators. Therefore, in the hands of experienced professionals, it is not only feasible but also a safe and effective method.

## Conclusions

The integration of hysteroscopic and laparoscopic approaches offers a conservative and minimally invasive treatment option that yields optimal surgical results.

In young and fertile women, the combination of hysteroscopy and laparoscopy is a promising and safe approach for correcting uterine scar defect along with RPOC. This approach enables simultaneous isthmoplasty and complete removal of placental remnants. The combined advantages of both techniques result in a more comprehensive defect correction and help preventing major complications. Nonetheless, further studies are required to establish standardisation of the technique and assess its potential impact on fertility and obstetric outcomes.

## Video scan (read QR)

https://vimeo.com/944865706/06797a7ada?share=copy

**Figure:**